# Tim‐3 blockade promotes iNKT cell function to inhibit HBV replication

**DOI:** 10.1111/jcmm.13600

**Published:** 2018-03-30

**Authors:** Yong Xu, Zehua Wang, Xianhong Du, Yuan Liu, Xiaojia Song, Tixiao Wang, Siyu Tan, Xiaohong Liang, Lifen Gao, Chunhong Ma

**Affiliations:** ^1^ Key Laboratory for Experimental Teratology of Ministry of Education Key Laboratory of Infection and Immunity of Shandong Province Department of Immunology School of Basic Medical Sciences Shandong University Jinan China

**Keywords:** HBV replication, iNKT cells, Tim‐3

## Abstract

Increased expression of T cell immunoglobulin and mucin domain‐3 (Tim‐3) on invariant natural killer T (iNKT) cells is reported in chronic hepatitis B virus (HBV) infection. However, whether Tim‐3 regulates iNKT cells in chronic HBV condition remains unclear. In this study, our results showed that the expression of Tim‐3 was up‐regulated on hepatic iNKT cells from HBV‐transgenic (Tg) mice or iNKT cells stimulated with α‐galactosylceramide (α‐Galcer). Compared with Tim‐3^−^ iNKT cells, Tim‐3^+^ iNKT cells expressed more IFN‐γ, IL‐4 and CD107a, indicating a strong relationship between Tim‐3 and iNKT cell activation. Constantly, treatment of Tim‐3 blocking antibodies significantly enhanced the production of IFN‐γ, TNF‐α, IL‐4 and CD107a in iNKT cells both in vivo and in vitro. This Tim‐3^−^ mediated suppression of iNKT cells was further confirmed in Tim‐3 knockout (KO) mice. Moreover, Tim‐3 blockade promoted α‐Galcer‐triggered inhibition of HBV replication, displaying as the decreased HBV DNA and HBsAg level in serum, and down‐regulated pgRNA expression in liver tissues. Collectively, our data, for the first time, demonstrated the potential role of Tim‐3 blockade in promoting iNKT cell‐mediated HBV inhibition. Therefore, combination of α‐Galcer with Tim‐3 blockade might be a promising approach in chronic hepatitis B therapy.

## 1 INTRODUCTION

Chronic hepatitis B (CHB) is a major public health burden, with an estimated of 250 million carriers worldwide.^1^, ^2^ Patients with CHB have a high risk of developing to hepatocellular carcinoma (HCC), which is the third leading cause of cancer mortality.^3^ Effective control of chronic HBV infection is an important step to prevent HCC. As reported, adaptive immunity especially HBV‐specific CD8^+^T cell‐mediated immunity plays a vital role in control of chronic HBV infection.^4^ However, the initial factor to start HBV‐specific adaptive immunity is less identified. According to recent reports, NKT cells played a key role in activating adaptive response.^5^ In the absence of NKT cell activation, adaptive immunity and HBV inhibition were largely diminished,^5^ which suggested that NKT cell might be an important checkpoint in HBV control.

NKT cells are an unconventional group of T cells that recognize lipid or glycolipid antigens.^6^ iNKT cells or type I NKT cells are a unique subset of NKT cells, defined by the expression of an invariant antigen receptor encoded by Vα14Jα18 (mouse) or Vα24Jα18 (human).^7^, ^8^ Specially, iNKT cells are CD1d‐restricted and can be activated by α‐Galcer, a glycolipid from marine sponge.^7^, ^8^ iNKT cells, on activation, can produce both Th1 cytokines such as IFN‐γ and Th2 cytokines such as IL‐4/IL‐13 within a few hours, the intensity and magnitude of which are closely related to various disease outcome such as cancer and infection.^9^, ^10^, ^11^, ^12^ Increasing evidence suggests that iNKT cells play a key role in anti‐HBV immunity.^13^, ^14^, ^15^ In a clinical research, the per cent of peripheral iNKT cells was decreased in patients with CHB, but antiviral treatment increased their frequencies,^16^, ^17^ which meant that iNKT cells might be linked to CHB development. The underlying mechanisms of iNKT cell involving in antiviral immune responses are further demonstrated by employing HBV mouse models, which showed that iNKT cells contributed to the priming of antiviral B and T cell immunity.^5^ α‐Galcer could promote the induction and proliferation of HBV‐specific cytotoxic T lymphocytes (CTLs).^18^ Moreover, α‐Galcer was proved to inhibit HBV replication by directly activating iNKT cells and by secondarily activating natural killer (NK) cells to secrete antiviral IFN‐γ or IFN‐α/β.^13^ Therefore, improving iNKT cell function using α‐Galcer seems a feasible strategy for clinical practice of curing patients with CHB. However, α‐Galcer used as a monotherapy resulted in an obvious decrease in iNKT cell number in patients with CHB and did not clearly affect HBV DNA level.^19^ Recent work from *Shi* laboratory showed that activating CD28/CD80 signal or blocking of programmed death (PD)‐1/PD‐L1, combined with α‐Galcer in HBV‐Tg mouse, had obtained a better control of HBV replication,^20^ strongly suggested that immune checkpoints might be new targets to reinforce iNKT cell function to inhibit HBV replication.

As a well‐known immune checkpoint, Tim‐3 has been widely studied in a variety of immune cells, including Th1 cells, CTLs and NK cells.^21^, ^22^ In these cells, Tim‐3 has been described of playing roles in the regulation of cell apoptosis, proliferation, cytotoxicity and cytokine production. But little is reported about the role of Tim‐3 on iNKT cells. So far as we know, Tim‐3 was highly expressed on peripheral NKT‐like (CD3^+^CD16/CD56^+^) cells in patients with rheumatoid arthritis or lung cancer^23^, ^24^ and was also elevated on NKT cells or NKT‐like (CD3^+^NK1.1^+^) cells in septic mice,^25^, ^26^ both of which indicating a possible relation between Tim‐3 and disease development. As to the regulation of Tim‐3 on iNKT cells, current researches showed that activating Tim‐3 pathway by binding to its ligand, galectin‐9 (Gal‐9), affected apoptosis of iNKT cells in various models.^25^, ^26^, ^27^ In the condition of CHB, published data reported a higher expression of Tim‐3 on peripheral NKT cells in patients with CHB,^28^ but the possible role of Tim‐3‐NKT axis in HBV control is still largely unknown.

Here, we studied the role of Tim‐3 on regulating iNKT cells in α‐Galcer‐induced acute hepatitis model in the background of HBs‐Tg C57BL/6 mice or HBV‐Tg Balb/c mice. Data showed that CD3^+^CD1d^+^iNKT cells were activated by α‐Galcer with an increased Tim‐3 expression, which was consistent with previous reports. Blocking Tim‐3 pathway with anti‐Tim‐3 neutralizing antibodies greatly promoted the ability of iNKT cells to produce cytokines and cytotoxic granules, which indicated a negative regulatory role of Tim‐3 on iNKT cells. This role was confirmed in Tim‐3 KO mice. Furthermore, Tim‐3 blockade significantly enhanced the HBV suppression induced by α‐Galcer. This may shed a light on future studies of iNKT cell and Tim‐3/iNKT cell‐based HBV immunotherapy.

## 2 MATERIALS AND METHODS

### 2.1 Mice and animal studies

Wild‐type 6‐ to 8‐week‐old male HBV‐Tg Balb/c (containing HBV whole genome, purchased from Infectious Disease Center of No. 458 Hospital, Guangzhou, China), HBs‐Tg C57BL/6 mice (containing partial HBV genome from the Vital River experimental animal company, Beijing, China) and Tim‐3 KO mice (prepared using TALEN strategy in C57BL/6 mice and supported by Sidansai Biotechnology Company, Shanghai, China) were housed in the Animal Facility under specific pathogen‐free conditions.

For acute hepatitis model, 2 μg of α‐Galcer or solvent control (0.1% DMSO in physiological saline) was tail‐vein injected into HBV‐Tg, HBs‐Tg or Tim‐3 KO mice. Mice were killed at 2 hours (for iNKT cells function assay) or 24 hours (for HBV evaluation) post‐injection. Serum was collected for alanine aminotransferase (ALT) and cytokines evaluation. Liver tissue was collected for paraffin sections and stained with haematoxylin and eosin (H&E). All procedures were approved by the Animal Care and Use Committee of Shandong University.

### 2.2 Preparation of intrahepatic lymphocytes

Intrahepatic lymphocytes (IHLs) were separated for functional testing. Briefly, mice livers were harvested, teased apart and mashed through a nylon mesh to get primary cell suspension. After lysing red blood cells in the suspension, IHLs were obtained by centrifugation over 40% Percoll solution (GE Healthcare, Uppsala, Sweden).^9^ Isolated IHLs were maintained with 10% FBS in 1640 medium (Gibco). For 2‐hour culture with brefeldin A (BFA, BioLegend), IHLs were harvested for flow cytometry (FCM).

### 2.3 Tim‐3 blocking assay

Tim‐3 in vivo blockade was performed by intraperitoneal (i.p.) injection of 100 μg of aTim‐3 (eBioscience) 24 hours before administration of α‐Galcer. For another 24 hours, mice were killed for experiments.

For blocking Tim‐3 pathway in iNKT cells, IHLs were pre‐incubated with 5 μg of anti‐Tim‐3 antibodies (eBioscience) for 30 minutes and then stimulated with α‐Galcer (1 μg/mL) for another 6 hours. BFA was added 4 hours before cell harvest.

### 2.4 α‐Galcer stimulation

Freshly separated IHLs were stimulated with α‐Galcer in different doses or at indicated time‐points. The expression of Tim‐3 or CD69 on iNKT cells was detected by FCM.

### 2.5 Flow cytometry

Anti‐mouse antibodies used here include CD1d tetramer (provided by the NIH Tetramer Facility), anti‐CD3, anti‐NK1.1, anti‐Dx5, anti‐NKG2D, anti‐NKG2A, anti‐CD69, anti‐IL‐4, anti‐IFN‐γ, anti‐TNF‐α and anti‐CD107a (from BioLegend) and anti‐Tim‐3 from eBioscience. Briefly, cell membrane markers were directly labelled for 30 minutes in dark at 4°C. For intracellular staining, freshly isolated IHLs were stimulated with or without α‐Galcer for 6 or 2 hours, and Brefeldin A (BFA) was added into the cell culture 4 or 2 hours before harvest. For detecting CD107a expression, fluorescence‐labelled anti‐CD107a was added into cell culture and maintained for the whole incubation process. All samples were tested by BD FACSAria III flow cytometer (BD Biosciences), and FCM data were analysed using BD FACSDiva 7.0 or FlowJo 7.0 software.

### 2.6 HBV evaluation

Peripheral blood was collected immediately when mice killed, and serum was prepared for detection. HBV DNA copies were quantified by real‐time PCR (Qiagen, Germany). ALT level was detected by ALT Kit (Nanjing Jiancheng Bioengineering Institute, China). HBsAg was quantified by Diagnostic Kit for HBsAg (ELISA) (InTec Products, INC, XIAMEN, China), and standard curve of HBsAg was made using standards from ProSpec‐Tany TechnoGene Ltd., Israel. IFN‐γ or TNF‐α level was detected using ELISA kit from DAKEWE, China. Mouse livers were also used to extract tissue RNA. The expression of viral pgRNA was measured or quantified by PCR or real‐time PCR (primer sequences: 5′‐3′ CTCAATCTCGGGAATCTCAATGT/AGGATAGAACCTAGCAGGCATAAT; products: 231 base pairs).^29^

### 2.7 Statistical analysis

Statistical analysis was performed with the paired or unpaired Student's *t* test using the GraphPad Prism 5 software. For all tests, *P* ⩽ .05 (∗), *P* ⩽ .01 (∗∗) or *P* ⩽ .001 (∗∗∗) was considered as significant.

## 3 RESULTS

### 3.1 Tim‐3 expression is up‐regulated on iNKT cells in HBV‐Tg mice and correlated with iNKT cell activation

**Figure 1:**
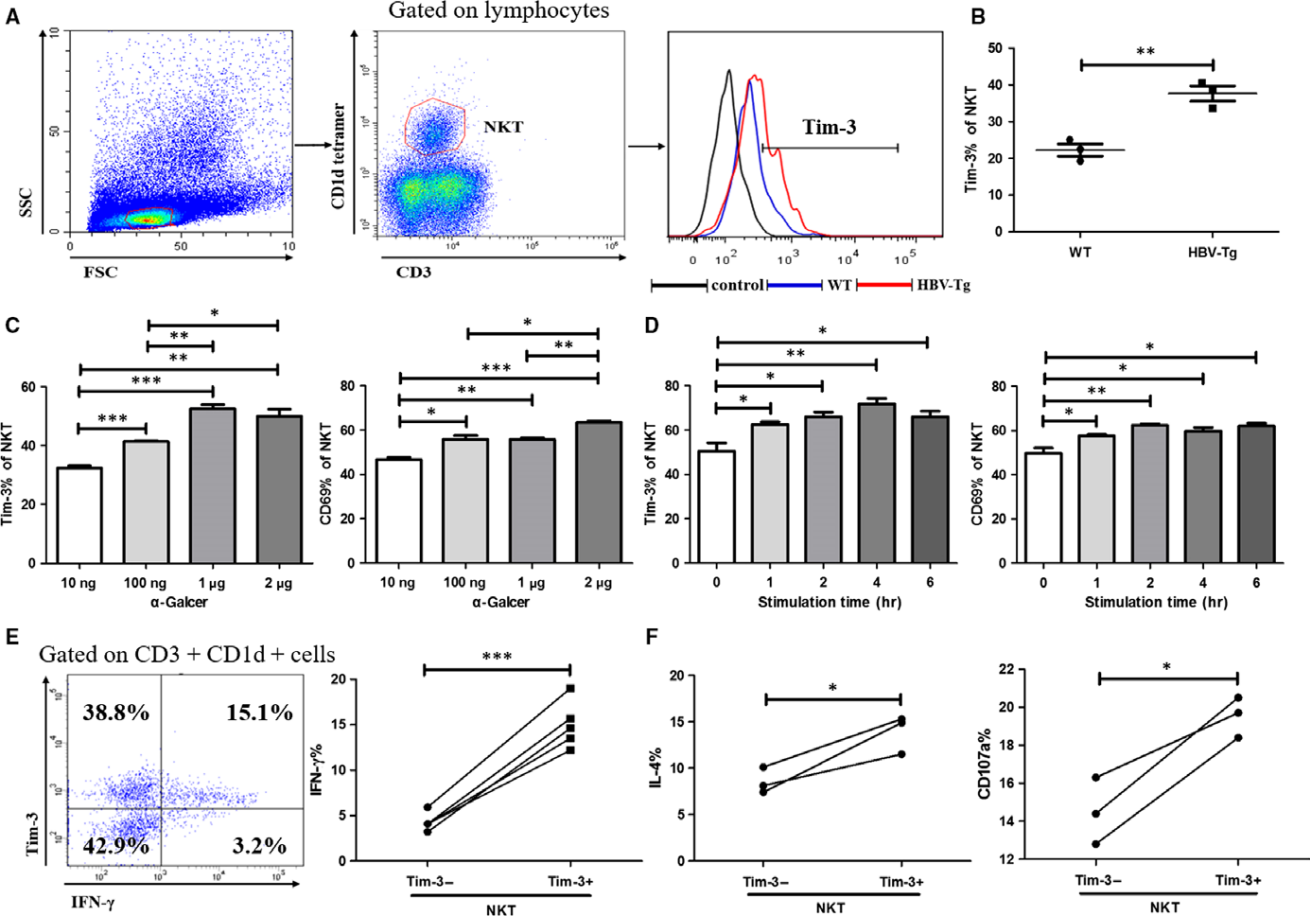
Tim‐3 expression was associated with iNKT cells activation. IHLs were separated and analysed by flow cytometry. Gated on CD3^+^ CD1d^+^ iNKT cells, the expression of Tim‐3 was analysed (A) and compared between HBV‐Tg mice and WT controls (B) (n = 3). IHLs were stimulated with α‐Galcer at different concentrations (C) or stimulated with 1 μg/mL α‐Galcer for indicated time (D), and the expression of Tim‐3 and CD69 on iNKT cells was analysed. IHLs were stimulated with 1 μg/mL α‐Galcer for 6 h, and the expression of IFN‐γ (E) (n = 5), IL‐4 and CD107a (F) (n = 3) in iNKT cells was detected and compared between Tim‐3^+^ iNKT and Tim‐3‐iNKT cells. Data were analysed using paired or unpaired Student's *t* test. For all graphs: *P* < .05 (∗), *P* < .01 (∗∗) or *P* < .001 (∗∗∗) was considered as significant

To investigate the role of Tim‐3 in iNKT cells in CHB, we firstly detected the expression of Tim‐3 on iNKT cells in HBV‐Tg mice. Gated on CD3^+^CD1d^+^cells, Tim‐3 expression of iNKT cells was analysed (Figure 1A). Comparing with control mice, we found membrane Tim‐3 level of iNKT cells was much higher in HBV‐Tg mice (Figure 1B). As Tim‐3 is expressed on activated T cells and NK cells, we came to evaluate the Tim‐3 expression on activated iNKT cells. As shown in Figure 1C, upon stimulation of α‐Galcer, the expression of both Tim‐3 and CD69 on iNKT cells was significantly up‐regulated in a concentration‐dependent manner. Meanwhile, under the stimulation of α‐Galcer, Tim‐3 and CD69 levels were elevated over time (Figure 1D). These data indicated that Tim‐3, like CD69, could be considered as a cell surface marker on activated iNKT cells. Constantly, FCM results demonstrated that, comparing with Tim‐3‐iNKT cells, Tim‐3^+^iNKT cells expressed more IFN‐γ (Figure 1E), as well as IL‐4 and CD107a (Figure 1F). Taken together, our data showed that membrane Tim‐3 on iNKT cells could be induced and was positively associated with iNKT cell active status.

### 3.2 Tim‐3 blockade promotes iNKT cell function

**Figure 2:**
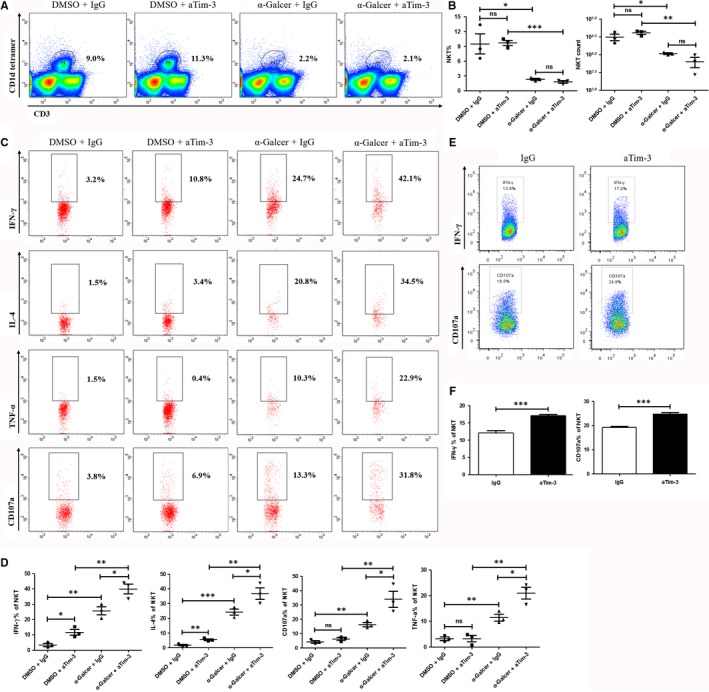
Tim‐3 blockade promoted iNKT cell function in HBV‐Tg mice. 100 μg of anti‐Tim‐3(aTim‐3) or IgG antibodies was i.p. injected into HBV‐Tg mice. 24 h later, 2 μg of α‐Galcer or DMSO control was i.v. injected. Another 24 h later, experimental mice were killed and IHLs were isolated and detected. The percentage and number of iNKT cells were compared between each group (A and B) (n = 3). Gated on CD3^+^ CD1d^+^ iNKT cells, the production of IFN‐γ, TNF‐α, IL‐4 and CD107a was detected and analysed (C and D) (n = 3). For in vitro assay, IHLs from HBV‐Tg mice were pre‐incubated with 5 μg/mL of anti‐Tim‐3(aTim‐3) or IgG antibodies for 30 minutes and then stimulated with 1 μg/mL α‐Galcer for 6 h. The expression of IFN‐γ and CD107a in iNKT cells was compared (E and F). All data were analysed using paired or unpaired Student's *t* test. For all graphs: ns (no significance), *P* < .05 (∗), *P* < .01 (∗∗) or *P* < .001 (∗∗∗)

**Figure 3:**
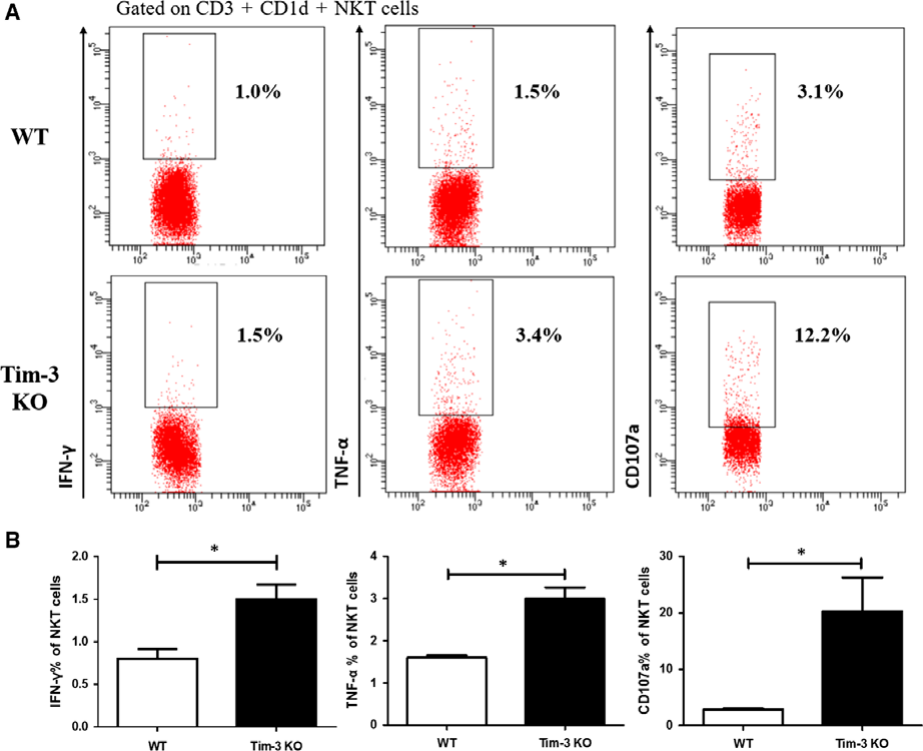
α‐Galcer promoted iNKT cell function in Tim‐3 KO mice. 2 μg of α‐Galcer was i.p. injected into Tim‐3 KO mice or control mice; 2 h later, these mice were killed and IHLs were separated. Functional tests showed that the production of IFN‐γ, TNF‐α and CD107a of iNKT cells was much higher in Tim‐3 KO mice (A and B) (n = 3). *P* < .05 (∗)

To further clarify the role of Tim‐3 in regulating iNKT cells, we blocked Tim‐3 pathway with 100 μg of anti‐Tim‐3 (aTim‐3) in α‐Galcer‐induced hepatitis model. FCM data showed that 2 μg of α‐Galcer largely decreased both the per cent and number of hepatic iNKT cells within 24 hours (Figure 2A,B) which were consistent with previous report.^12^ Comparing with non‐α‐Galcer group, α‐Galcer strikingly increased the expression of IFN‐γ, TNF‐α, IL‐4 and CD107a in iNKT cells (Figure 2C,D). Specially, combination of α‐Galcer with anti‐Tim‐3 promoted iNKT cells to produce the most level of inflammatory cytokines and CD107a (Figure 2C,D). Interestingly, in the absence of α‐Galcer, Tim‐3 blockade alone could also improve iNKT cell function in aspects of IFN‐γ and IL‐4 (Figure 2C,D). This modulatory effect of Tim‐3 in iNKT cells was verified with in vitro studies. Pre‐incubation with 5 μg/mL of anti‐Tim‐3 enhanced the expression of IFN‐γ and CD107a in CD3^+^CD1d^+^iNKT cells from naive mice (Figure 2E,F), indicating Tim‐3 as a potential negative regulator affecting iNKT cell function. The role of Tim‐3 in iNKT cells was further confirmed using Tim‐3 KO mice. As shown in Figure 3, comparing with control mice, α‐Galcer promoted iNKT cell functions in Tim‐3 KO mice in a very short time (Figure 3A,B). Taken together, our data suggested that Tim‐3 negatively modulated iNKT cell function in our animal model.

### 3.3 Tim‐3 blockade improves NK and CD8^+^T cell function

**Figure 4:**
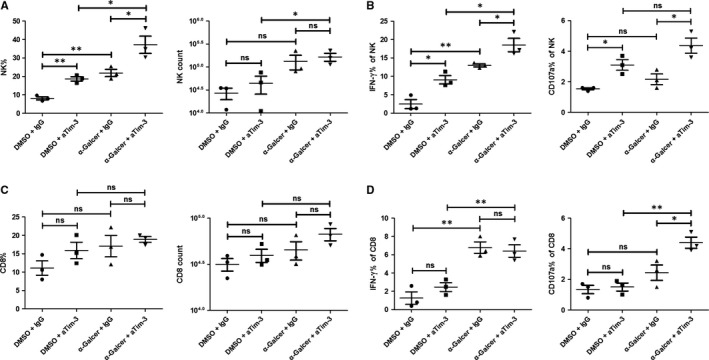
Tim‐3 blockade improved NK and CD8^+^ T cell function. In vivo assay was carried on as above described. The percentage and number of CD3^−^ Dx5^+^ NK cells (A) or CD3^+^ CD8^+^T cells (C) were analysed and compared (n = 3). The expression of IFN‐γ and CD107a on NK cells (B) or CD8^+^T (D) cells was also analysed (n = 3). All data were analysed using unpaired Student's *t* test. For all graphs: ns (no significance), *P* < .05 (∗); *P* < .01(∗∗)

iNKT cells are unique regulatory cells which secrete a large amount of cytokines and bridge innate and adaptive immunity.^6^ We then evaluated the role of α‐Galcer in regulating other immune cells including NK and CD8^+^T cells, which were reported to have critical roles in chronic HBV infection. As shown in Figure 4, α‐Galcer augmented the expression of IFN‐γ or CD107a in NK cells as well as CD8^+^T cells (Figure 4B,D). At the same time, the percentage of NK cells was increased (Figure 4A), which was consistent with previous reports.^13^ But α‐Galcer did not obviously impact CD8^+^T cells frequency (Figure 4C). Moreover, Tim‐3 blockade greatly improved α‐Galcer‐induced CD107a production from both NK cells and CD8^+^T cells (Figure 4B,D), which may assist iNKT cells to inhibit HBV replication. Especially for NK cells, which was reported as the main cell type of inhibiting HBV replication in HBV‐Tg mice, Tim‐3 blockade greatly enhanced their frequency and function (Figure 4A,B) in our model.

### 3.4 Tim‐3 blockade augments α‐Galcer‐induced inhibition on HBV replication

**Figure 5:**
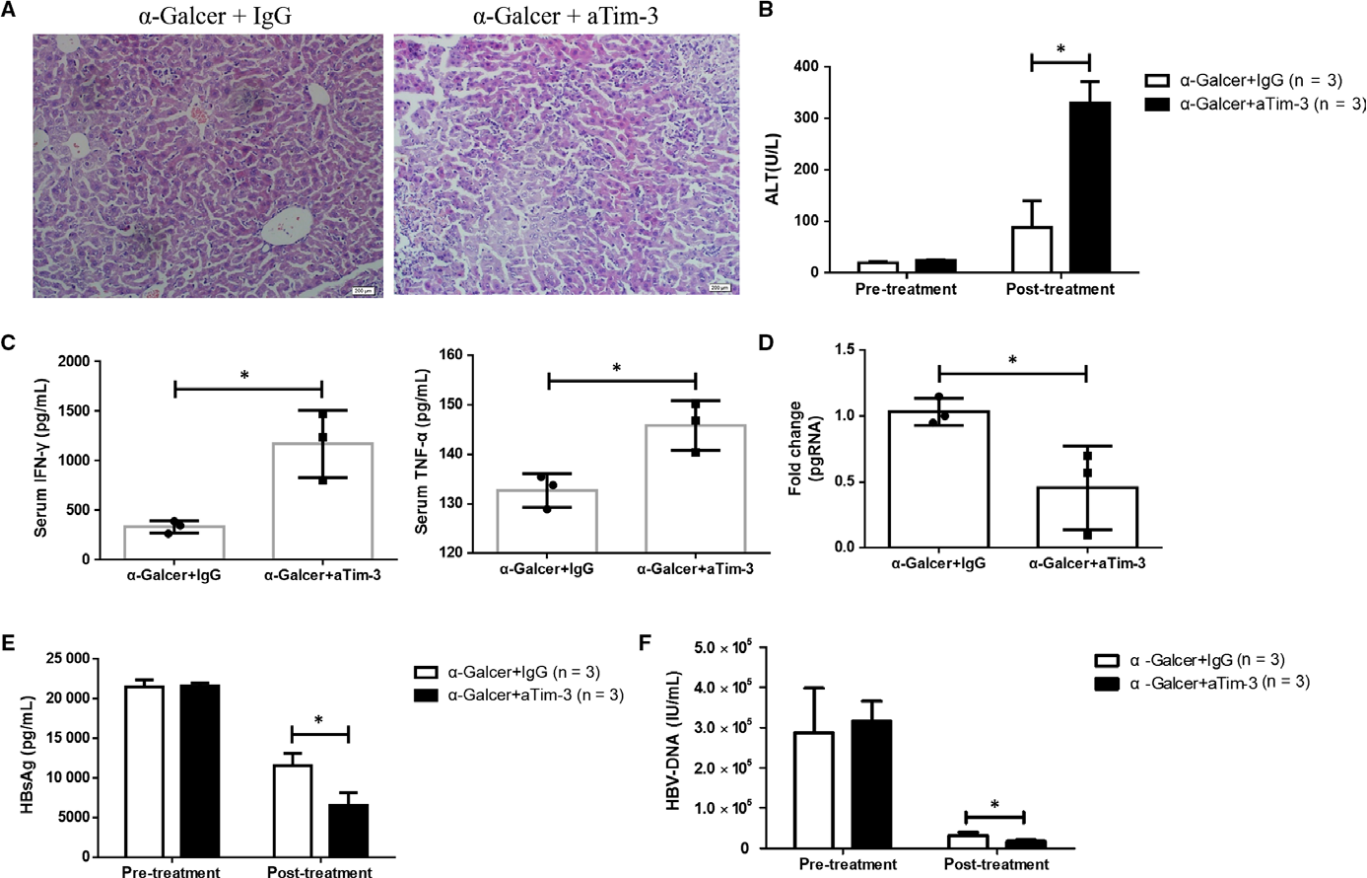
Tim‐3 blockade augmented α‐Galcer‐induced HBV inhibition. Acute hepatitis was induced in HBV‐Tg mice with 2 μg of α‐Galcer, and liver tissues were taken and stained with H and E at 24 h after treatment of α‐Galcer (A). Serum ALT (B), IFN‐γ, TNF‐α (C) and HBsAg (E) were detected and quantified by ELISA kits and compared with the level of pre‐treatment of α‐Galcer (n = 3). Serum HBV DNA copies (F) and the expression of pgRNA in liver tissues were detected by qPCR (D) (n = 3). All data were analysed using unpaired Student's *t* test. For all graphs: *P* < .05 (∗)

It has been previously reported that α‐Galcer inhibits HBV replication in HBV‐Tg mice.^13^ To address whether Tim‐3 promotes α‐Galcer‐initiated HBV inhibition, HBV‐Tg mice were treated with α‐Galcer alone (α‐Galcer +IgG) or α‐Galcer combined with anti‐Tim‐3 (α‐Galcer+aTim‐3). As expected, comparing with control group, mice from the group of α‐Galcer+aTim‐3 showed larger areas of necrosis in the liver (Figure 5A). Accordingly, serum ALT level was also higher in mice treated with α‐Galcer plus anti‐Tim‐3 (Figure 5B), together with increased concentration of IFN‐γ and TNF‐α (Figure 5C), which indicated that Tim‐3 blocking could promote α‐Galcer‐mediated inflammatory responses. More importantly, comparing with α‐Galcer control, Tim‐3 blockade further decreased serum level of HBsAg and HBV DNA copies in HBV‐Tg mice (Figure 5E,F). Furthermore, results of qPCR and PCR (Figure S1) showed that blocking Tim‐3 pathway inhibited pgRNA expression in HBV‐Tg mice (Figure 5D). Taken together, our data demonstrated that Tim‐3 blockade significantly enhances α‐Galcer‐mediated inhibition of HBV replication in vivo.

### 3.5 Tim‐3 blockade also promotes iNKT cell function to inhibit HBsAg secretion in HBs‐Tg mice

**Figure 6:**
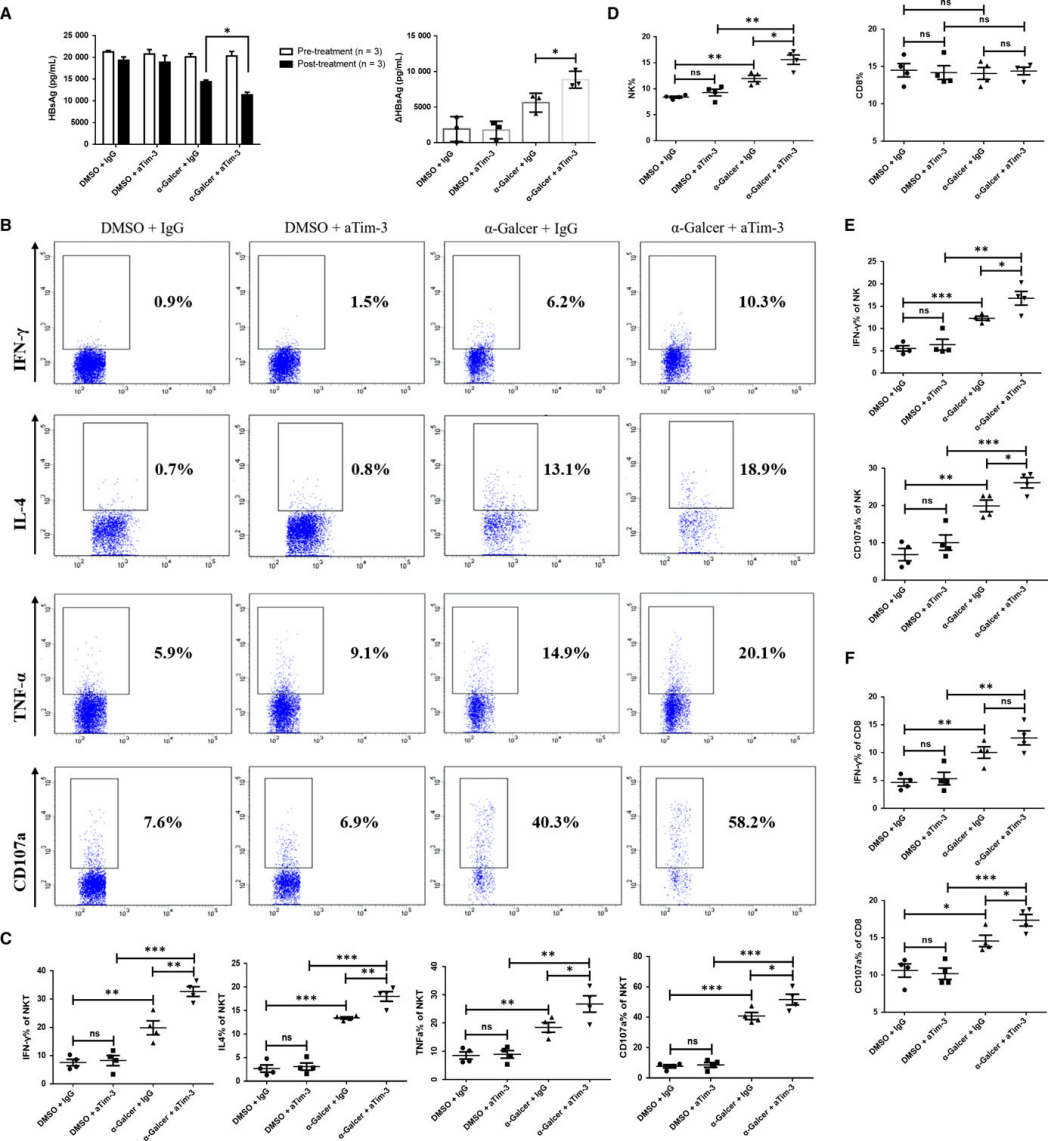
Tim‐3 blockade also promoted iNKT cell function to inhibit HBsAg secretion in HBs‐Tg mice. Experimental mouse model was established in HBs‐Tg mice and grouped as described in the Materials and Methods. Serum HBsAg level of pre‐/post‐treatment of α‐Galcer was quantified with ELISA kit (A, left panel), and the reduction in HBsAg level (pre‐treatment minus post‐treatment) was also analysed (A, right panel). Gated on CD3^+^ CD1d^+^population, the expression of IFN‐γ, TNF‐α, IL‐4 or CD107a of iNKT cells was analysed (B and C). NK cell or CD8^+^T cell frequency was compared among all groups (D), and cell functions were evaluated in aspects of IFN‐γ and CD107a expression (E and F). All data were analysed using unpaired Student's *t* test. For all graphs: ns (no significance), *P* < .05 (∗), *P* < .01 (∗∗) or *P* < .001 (∗∗∗)

To exclude the potential species specificity in Tim‐3 blockade promoted enhancement of iNKT cells and HBV inhibition, similar experiments were carried on in HBs‐Tg C57BL/6 mice. As shown in Figure 6A, after treatment with α‐Galcer, serum HBsAg level was largely decreased, and Tim‐3 blockade promoted α‐Galcer‐induced HBsAg reduction. Consistent with data from HBV‐Tg Balb/c mice, α‐Galcer could also promote the expression of IFN‐γ, TNF‐α, IL‐4 and CD107a in iNKT cells. These functions of iNKT cells were further augmented by engaging anti‐Tim‐3 (Figure 6B,C). Moreover, Tim‐3 blockade also improved NK and CD8^+^T cells to produce more IFN‐γ or CD107a (Figure 6E,F). Meanwhile, NK cell number was also increased after Tim‐3 blocking (Figure 6D). Collectively, Tim‐3 blockade promoted α‐Galcer‐induced HBV inhibition in HBs‐Tg mice which might be mediated by directly improving iNKT cell function and by indirectly improving NK cell function.

## 4 DISCUSSION

A major problem of chronic HBV infection is immune tolerance to HBV,^30^ thus how to break immune tolerance is a critical issue in developing new and effective therapies. CHB is characterized by massive dysfunction of immune cells majorly including HBV‐specific CD8^+^T cells and NK cells.^31^ One of the reasons leading to immune dysfunction is the expression of negative regulatory molecules, such as CTLA‐4, PD‐1 and IL‐10.^32^, ^33^ Tim‐3 is a newly identified molecule which has been reported playing important roles in regulating T cells, NK cells and macrophages. In some cases, like PD‐1, Tim‐3 was regarded as an exhaustion marker indicating damaged immune response, and blocking Tim‐3 pathway partly restored T/NK cells function.^34^, ^35^ Therefore, Tim‐3 was considered as an important checkpoint in disease development. Our work here highlights the potential of Tim‐3 blockade in immunotherapy not only for enhancing CD8^+^T/NK cells but also for iNKT cells.

In our work, we detected increased membrane Tim‐3 expression on iNKT cells in HBV‐Tg mice. Blocking Tim‐3 pathway significantly improved iNKT cell function, and this inhibitory role was further confirmed in Tim‐3 KO mice. More importantly, Tim‐3 blockade greatly enhanced α‐Galcer‐induced HBV suppression in vivo. As far as we know, this is the first report demonstrating the potential role of Tim‐3 blockade in promoting iNKT cell‐mediated HBV inhibition. But the molecular mechanism of Tim‐3 suppressing iNKT cells yet to be identified. Tim‐3 has been reported to associate with interleukin inducible T cell kinase (ITK) with its cytoplasmic tail which contains a conserved tyrosine residue (Y265) in T cells.^36^ Subsequent studies found Tim‐3 expression could regulate NF‐κB and NFAT reporter activity,^37^, ^38^ but these results were controversial and limited to T cell system. Whether Tim‐3 regulates iNKT cell function via affecting downstream Fyn or PI‐3K subunit needs further demonstration.^37^

Tim‐3 not only regulates iNKT cells function but also correlates with their activation status. After stimulating with α‐Galcer, Tim‐3 expression was increased on iNKT cells in both time and dosage‐dependent manner. The same trend was observed in CD69, an activation marker for immune cells. These data suggested that Tim‐3 was associated with iNKT cell activation. Moreover, functional tests showed that Tim‐3^+^iNKT cells had a higher expression of IFN‐γ, IL‐4 and CD107a. Collectively, above results gave us a hint that Tim‐3 expression might reflect the activation status of iNKT cells. However, Tim‐3^+^NKT cells appeared to be more sensitive to activation‐induced cell death. Since after α‐Galcer stimulation, the per cent of peripheral NKT cells was decreased, accompanied with high level of Tim‐3.^25^, ^27^ Consistent with that, we also found that Tim‐3^+^iNKT cells had a higher expression of annexin‐V (Figure S2). These results may suggest that Tim‐3 tends to play a role in the homeostasis of iNKT cells. However, further studies are demanded to uncover the underlying mechanisms of Tim‐3 modulating iNKT cells, which will be benefit for exploring the potential of iNKT cells in immunotherapy.

In the model of Tim‐3 blockade promoting α‐Galcer‐induced HBV inhibition, other immune cells, majorly including NK and CD8^+^T cells, were involved and played a critical role in this system. Activated iNKT cells can secrete large amounts of IFN‐γ/TNF‐α/CD107a, but due to α‐Galcer induced strikingly decrease in iNKT cell number, the overall antiviral effect of α‐Galcer on iNKT cells is doubtful. Thus, the subsequent activation of NK/CD8^+^T cells owing to IFN‐γ becomes the most powerful forces against HBV replication.^13^ Even though NK/CD8 T cells will take the place of overactivated iNKT cells, a randomized placebo‐controlled trial still failed to detect the success inhibition of HBV DNA in α‐Galcer‐treated patients with CHB, which we had to attribute this failure to the poor tolerance of α‐Galcer or the obvious drop of iNKT cell number.^19^ Constantly, we found α‐Galcer largely reduced the percentage of hepatic iNKT cells as well as the cell number in the mice models. But Tim‐3 blockade promoted IFN‐γ or CD107a production from CD8^+^T cells or NK cells and could also regulate NK cell frequency.^13^ It seems that the role of aTim‐3 promoting NK cells was independent of α‐Galcer, for we observed partly up‐regulated NK cell function in control group, the reason of which may be related to the mild inflammatory environment in liver tissues caused by control vehicle. Besides, another fact is that α‐Galcer‐induced HBV inhibition was abolished if iNKT cells were eliminated^13^; hence, we noticed that anti‐Tim‐3‐mediated HBV suppress was largely weakened when pre‐treating with anti‐PK136 antibodies in vivo, an antibody reacting with cell surface NK1.1 on NK/NKT cells (data not show). But these data may also have indicated the important role of CD8^+^T cells in our model and all need extra data to clarify the exact mechanisms. In conclusion, our data here suggested the involvement of CD8^+^T cells and NK cells in aTim‐3 boosted α‐Galcer‐initiated HBV inhibition.

Collectively, elevated Tim‐3 expression has been well identified on Th1 cells, CTLs, NK and NKT cells during HBV infection, and blocking Tim‐3 pathway restores cell functions in multiple dimensions. Hence, Tim‐3 expression is closely related to the development or prognosis of HBV‐related diseases. Here, in our work, we clarified the role of Tim‐3 on regulating cytokine production and cytotoxicity of iNKT cells. Blocking Tim‐3 pathway promoted the α‐Galcer‐mediated inhibition on HBV replication. In conclusion, Tim‐3 may be a breach for breaking immune tolerance of CHB infection partly via regulating iNKT cells.

## CONFLICT OF INTERESTS

No competing interests exist.
