# Supplementary material for: Tim‐3 blockade promotes iNKT cell function to inhibit HBV replication
Source: J Cell Mol Med. 2018 Mar 30;22(6):3192–201. doi: 10.1111/jcmm.13600 (PMC5980221; doi:10.1111/jcmm.13600)
Supplement: Supplementary file 1 [file JCMM-22-3192-s001.docx]

**Supplementary data to:**

Tim-3 blockade promotes iNKT cell function to inhibit HBV replication

Yong Xu, Zehua Wang, Xianhong Du, Yuan Liu, Xiaojia Song, Tixiao Wang, Siyu Tan, Xiaohong Liang, Lifen Gao, Chunhong Ma


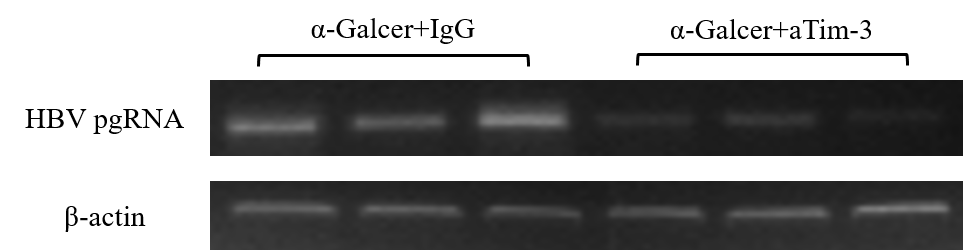


Figure 1. Tim-3 blockade promoted the control of HBV replication induced by α-Galcer. 100ug of aTim-3 were i.p. injected into HBV-Tg mice 24 hours before administration of α-Galcer. And 24 hours post α-Galcer treatment, mice were sacrificed and liver tissues were isolated for experiments. The expression of HBV pgRNA was detected using normal PCR and compared between IgG and aTim-3 groups (primer sequences: 5’-3’ CTCAATCTCGGGAATCTCAATGT /AGGATAGAACCTAGCAGGCATAAT; products: 231 base pairs).


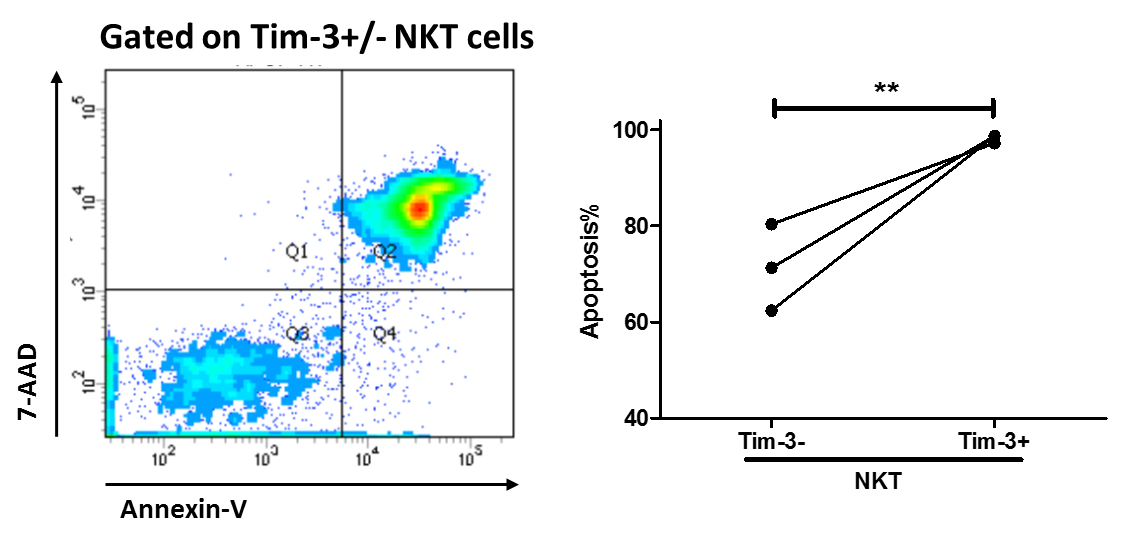


Figure 2. Tim-3 expression on NKT cells was correlated with apoptosis. IHLs were isolated as previous reported and stimulated with α-Galcer (1ug/ml) for 6 hours in vitro. Gated on Tim-3+/Tim-3-NKT cells, the expression of annexin-v was analyzed and compared. Data was analyzed using paired Student’s t test. p<0.01 (∗∗) was considered as significant.
